# The administration sequences of immune checkpoint inhibitors and chemotherapy cause discrete efficacy when treating non-small cell lung cancer: a retrospective study

**DOI:** 10.3389/fimmu.2025.1579420

**Published:** 2025-04-28

**Authors:** Bicheng Zhang, Yuxiao Song, Qian Min, Weiting Cheng, Jun Wang, Yang Fu, Jiaxin Yin

**Affiliations:** ^1^ Cancer Center, Renmin Hospital of Wuhan University, Wuhan, China; ^2^ Department of Oncology, The First Affiliated Hospital of Shandong First Medical University, Jinan, China; ^3^ Department of Oncology and Hematology, Xiangyang Hospital, Hubei University of Chinese Medicine, Xiangyang, China

**Keywords:** immune checkpoint inhibitors, chemotherapy, administration sequence, efficacy, non-small cell lung cancer

## Abstract

**Background:**

Immune checkpoint inhibitors (ICIs) combined with chemotherapy have become a standard first-line treatment for advanced non-small cell lung cancer (NSCLC). However, the optimal sequence of administrating the two treatments remains controversial.

**Methods:**

This study included advanced NSCLC patients who received ICIs combined with chemotherapy at Renmin Hospital of Wuhan University and Xiangyang Hospital, Hubei University of Chinese Medicine between 1st September 2020 and 30th September 2024. Patients were categorized into the concurrent, immune-chemo, and chemo-immune groups based on different sequences of treatment administration. The primary endpoints evaluated were survival and treatment efficacy. The secondary endpoint assessed was treatment-related adverse events (TRAEs).

**Results:**

This two-center, retrospective study included 270 NSCLC patients who received ICIs plus chemotherapy. Survival analysis revealed statistically significant differences across treatment groups. The median overall survival (mOS) durations were 636 days (concurrent group), 615 days (immune-chemo group), and 749 days (chemo-immune group), with a log-rank test demonstrating significant intergroup differences (*P* = 0.0017). Similarly, median progression-free survival (mPFS) showed distinct patterns at 178 days, 180 days, and 216 days for the respective groups (log-rank *P* = 0.0134). Additionally, the objective response rates (ORRs) for the three groups were 55.82% (72/129), 58.21% (39/67), and 68.92% (51/74), respectively. The incidence of TRAEs of any grade in the concurrent, the immune-chemo, and the chemo-immune groups was 77.52% (100/129), 65.67% (44/67), and 59.46% (44/74) rates, respectively, which was a significant difference (χ²=7.91, *P*=0.019). Despite patients experiencing Grade 3 or higher TRAEs had extremely poor prognoses, overall, patients who developed any grade of TRAEs had better survival outcomes, particularly those with skin or endocrine toxicity.

**Conclusions:**

These findings suggest that the administration sequence of chemotherapy followed by ICIs may yield the greatest clinical benefit, providing a basis for clinical decision-making.

## 1 Introduction

Non-small cell lung cancer (NSCLC) is a highly prevalent and deadly form of cancer, with persistently high annual incidence and mortality rates worldwide (1). Immune checkpoint inhibitors (ICIs) have revolutionized the clinical management of NSCLC patients (2, 3). They improve anti-tumor T-cell immunity mainly by blocking the interaction of the coinhibitory receptor programmed cell death-1 (PD-1) with its main ligand programmed death-ligand 1 (PD-L1) (4, 5). However, only a small proportion of patients can benefit from ICIs monotherapy (6). Consequently, various combination strategies have been designed to augment the treatment efficacy, such as ICIs plus chemotherapy, ICIs plus radiotherapy, and dual immunotherapy (7–9).

The high anti-tumor efficacy of ICIs plus chemotherapy has been widely confirmed. *In vitro* experiments have revealed that chemotherapy augmented systemic intratumor immune responses through tumor intrinsic mechanisms including releasing tumor antigens, modulating tumor-infiltrating lymphocytes, and upregulating the expression of PD-L1 (10–12). Numerous phase III randomized controlled trials (RCTs) have also demonstrated that ICIs plus chemotherapy significantly improved survival outcomes in NSCLC patients (13–15). Therefore, ICIs plus chemotherapy has been approved as the standard first-line treatment for advanced NSCLC patients and is widely applied in clinical practice (16, 17). However, the optimal sequence of ICIs and chemotherapy has not yet been fully explored.

In clinical practice, two predominant perspectives regarding the integration of ICIs with chemotherapy. One group of scholars follows the protocol established by RCTs, advocating for the concurrent administration of ICIs and chemotherapy on the same day (17). The other group supports a sequential therapy approach, positing that the orderly application of the two treatment strategies can enhance therapeutic efficacy while reducing adverse effects (18). In addition, sequential therapy is divided into two approaches within the clinical context. One is chemo-immunotherapy, which involves the pre-treatment with chemotherapy followed by ICIs, the other is immuno-chemotherapy, where ICIs are administered before chemotherapy. However, there is no definitive evidence or guidelines specifying the sequence of the two treatments currently. To address this clinical question, we conducted a retrospective study to explore the optimal sequence of ICIs plus chemotherapy. Overall survival (OS), progression-free survival (PFS), and objective response rate (ORR) were utilized to assess the efficacy, while treatment-related adverse events (TRAEs) were employed to evaluate the safety.

## 2 Materials and methods

### 2.1 Study design

The study adhered to the principles outlined in the Declaration of Helsinki. This retrospective trial received approval from the Clinical Research Ethics Committee of Renmin Hospital of Wuhan University (ID number: WDRM2024-K252), and the patients or their guardians all signed informed consent before enrollment. All patient data collected during follow-up adhered to relevant data protection and privacy regulations.

### 2.2 Inclusion and exclusion criteria

This study included NSCLC patients who were treated at Renmin Hospital of Wuhan University and Xiangyang Hospital, Hubei University of Chinese Medicine between 1st September 2020 and 30th September 2024. The following are the inclusion criteria: (a) patients with histologically confirmed advanced NSCLC (according to the 8th edition TNM staging, diagnosed as stage III B to IV); (b) patients treated with ICIs plus chemotherapy as first-line treatment; (c) at least one primary or metastatic lesion that can be measured or evaluated by imaging data, as well as relevant imaging data for measurement and evaluation; and (d) clinical and pathological data are complete. The exclusion criteria were as follows: (a) patients treated with any other drugs combined with ICIs and chemotherapy (such as antiangiogenic drugs); (b) patients treated with ICIs plus chemotherapy for less than two cycles; and (c) patients with non-squamous NSCLC harboring sensitive gene mutations such as *EGFR*, *ALK*, and *ROS1*, *etc.*

### 2.3 Data collection

Patients’ clinical and survival data were collected retrospectively, including age, gender, smoking history, Eastern Cooperative Oncology Group-Performance Status (ECOG-PS) score, histological type, clinical stage, treatment line, metastatic site, PD-L1 expression, treatment information, OS, PFS, ORR, and safety profile. NSCLC patients were stratified into three cohorts based on the chronological sequence of treatment administration: (1) the concurrent group, receiving ICIs combined with chemotherapy agents on the same day; (2) the immune-chemo group, administered ICIs followed by chemotherapy initiation after an interval of more than 24 hours; and (3) the chemo-immune group, which commenced chemotherapy first and then received ICIs after a 24-hour interval. OS was calculated as the time from the initiation of treatment to any-cause mortality. PFS was delineated as the duration from the commencement of combination therapy to the onset of disease progression or death from any cause. Patients were assessed for efficacy every two treatment cycles until death. The clinical response was evaluated as complete response (CR), partial response (PR), and stable disease (SD) according to the Response Evaluation Criteria in Solid Tumors Version 1.1 (RECIST v.1.1) and iRECIST. The ORR was calculated as the ratio of CR plus PR, while the disease control rate (DCR) was the percentage of patients with ORR plus SD. The severity of TRAEs was graded according to the National Cancer Institute Common Terminology Criteria for Adverse Events, version 4.0. All patients were followed up by medical records review and telephone counseling.

### 2.4 Statistical analysis

The group comparisons of count data were executed using the chi-square test or Fisher’s exact test. We estimated the survival curves of OS and PFS using the Kaplan-Meier method, and differences were compared using the Log-rank test. The hazard ratio (HR) and 95% confidence interval (CI) were estimated using the Cox proportional hazard regression models. All statistical analyses were conducted using SPSS 26.0 and GraphPad Prism 9. A *P* value < 0.05 was deemed statistically significant.

## 3 Results

### 3.1 Patients characteristics

**Figure 1:**
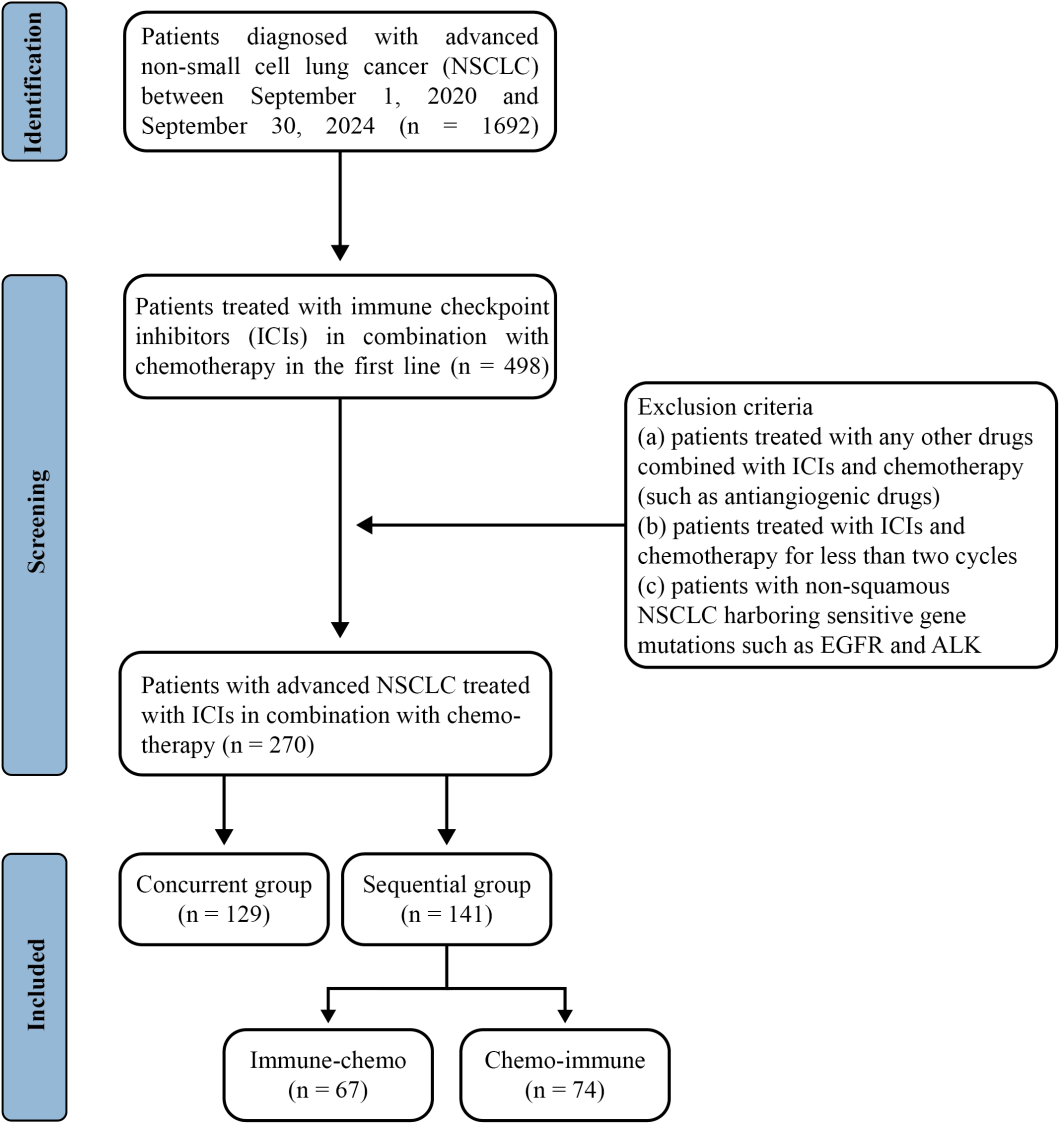
Flowchart of enrollment and grouping in this retrospective study.

Following a meticulous screening process (**Figure 1**), 270 NSCLC patients from the two centers treated with ICIs plus chemotherapy were included in the present study. The baseline patient demographics and clinicopathological characteristics are summarized in **Table 1**. In the total population, the median age was 62 (range: 29-88) years, with a higher proportion of male patients (207 cases, 76.7%); 115 (42.6%) patients had a smoking history and 235 (87.0%) patients had an ECOG-PS score of 1. Adenocarcinoma was the predominant histological subtype (176 cases, 65.2%) and most patients (197 cases, 73.0%) were diagnosed with stage IV at the initiation of treatment. 58 patients (21.5%) had brain metastases, 31 patients (11.5%) had liver metastases, and 84 patients (31.1%) had bone metastases. Among the cohort, 69 (25.6%) patients were found to have negative PD-L1 expression [tumor proportion score (TPS) < 1%], while 127 (47.0%) patients were PD-L1 positively expressed (TPS ≥ 1%). All patients were treated with PD-1 inhibitors plus chemotherapy, with the latter being platinum-based regimens regardless of whether squamous or adenocarcinomas.

**Table 1:** Demographics and clinicopathological characteristics of advanced NSCLC patients in the study cohort.

Characteristics	Renmin Hospital Cohort (n = 189)	Xiangyang Hospital Cohort (n = 81)	Total (n = 270)	Statistic	*P* value
Gender				χ²=4.69	0.030
Male	138 (73.0%)	69 (85.2%)	207 (76.7%)		
Female	51 (27.0%)	12 (14.8%)	63 (23.3%)		
Age (years)				χ²=1.81	0.405
< 65	113 (59.8%)	42 (51.9%)	155 (57.4%)		
≥ 65	76 (40.2%)	39 (48.1%)	115 (42.6%)		
≥ 75	15 (7.9%)	6 (7.4%)	21 (7.8%)		
ECOG-PS				χ²=1.77	0.412
0	8 (4.2%)	1 (1.2%)	9 (3.3%)		
1	162 (85.7%)	73 (90.1%)	235 (87.0%)		
2	19 (10.1%)	7 (8.7%)	26 (9.7%)		
Histological subtype				χ²=2.73	0.256
Adenocarcinoma	128 (67.7%)	48 (59.2%)	176 (65.2%)		
Squamous carcinoma	49 (25.9%)	29 (35.8%)	78 (28.9%)		
Others	12 (6.4%)	4 (5.0%)	16 (5.9%)		
Smoking status				χ²=3.05	0.081
Never-smokers	115 (60.8%)	40 (49.4%)	155 (57.4%)		
Former or current smokers	74 (39.2%)	41 (50.6%)	115 (42.6%)		
Clinical stage				χ²=5.58	0.018
IIIB-C	59 (31.2%)	14 (17.3%)	73 (27.0%)		
IV	130 (68.8%)	67 (82.7%)	197 (73.0%)		
PD-L1 expression				χ²=2.16	0.339
TPS < 1%	53 (28.0%)	16 (19.7%)	69 (25.6%)		
TPS ≥ 1%	87 (46.0%)	40 (49.4%)	127 (47.0%)		
Unknown	49 (26.0%)	25 (30.9%)	74 (27.4%)		
Metastatic sites					
Brain	44 (23.3%)	14 (17.3%)	58 (21.5%)	χ²=1.21	0.272
Liver	19 (10.1%)	12 (14.8%)	31 (11.5%)	χ²=1.27	0.261
Bone	56 (29.6%)	28 (34.6%)	84 (31.1%)	χ²=0.65	0.422
Immunotherapy regime				χ²=4.96	0.174
Tislelizumab	88 (46.6%)	40 (49.4%)	128 (47.4%)		
Pembrolizumab	30 (15.9%)	16 (19.8%)	46 (17.0%)		
Sintilimab	29 (15.3%)	16 (19.8%)	45 (16.7%)		
Camrelizumab	42 (22.2%)	9 (11.1%)	51 (18.9%)		
Treatment sequence				χ²=2.01	0.366
Concurrent group	85 (45.0%)	44 (54.3%)	129 (47.8%)		
Immune-chemo group	49 (25.9%)	18 (22.2%)	67 (24.8%)		
Chemo-immune group	55 (29.1%)	19 (23.5%)	74 (27.4%)		

χ², Chi-square test; ECOG-PS, Eastern Cooperative Oncology Group performance status; TPS, tumor proportion score.

Total patients were categorized into three groups based on the administration sequence of ICIs in combination with chemotherapy. The concurrent group comprised 129 patients, the immune-chemo group included 67 patients, and the chemo-immune group consisted of 74 patients. **Table 2** presented the demographic and clinicopathological features of the three patient cohorts, which were largely consistent with the overall patient population.

**Table 2:** Demographics and clinicopathological characteristics were stratified by the sequence of ICIs plus chemotherapy in three groups.

Characteristics	Concurrent group (n = 129)	Immune-chemo group (n = 67)	Chemo-immune group (n = 74)	Statistic	*P* value
Gender				χ²=2.33	0.312
Male	102 (79.1%)	53 (79.1%)	52 (70.3%)		
Female	27 (20.9%)	14 (20.9%)	22 (29.7%)		
Age				χ²=5.84	0.212
< 65	70 (54.3%)	46 (68.7%)	39 (52.7%)		
≥ 65	59 (45.7%)	21 (31.3%)	35 (47.3%)		
≥ 75	13 (10.0%)	3 (4.5%)	5 (6.8%)		
ECOG-PS				–	0.158
0	7 (5.4%)	2 (2.9%)	0 (0%)		
1	113 (87.6%)	58 (86.7%)	64 (86.5%)		
2	9 (7.0%)	7 (10.4%)	10 (13.5%)		
Histological subtype				–	0.935
Adenocarcinoma	87 (67.4%)	42 (62.7%)	47 (63.5%)		
Squamous carcinoma	34 (26.4%)	21 (31.3%)	23 (31.1%)		
Others	8 (6.2%)	4 (6.0%)	4 (5.4%)		
Smoking status				χ²=12.08	0.002
Never-smokers	60 (46.5%)	46 (68.7%)	49 (66.2%)		
Former or current smokers	69 (53.5%)	21 (31.3%)	25 (33.8%)		
Clinical stage				χ²=5.99	0.050
IIIB–C	26 (20.2%)	23 (34.3%)	24 (32.4%)		
IV	103 (79.8%)	44 (65.7%)	50 (67.6%)		
PD-L1 expression				χ²=23.20	<.001
TPS < 1%	22 (17.1%)	16 (23.9%)	31 (41.9%)		
TPS ≥ 1%	77 (59.7%)	29 (43.3%)	21 (28.4%)		
Unknown	30 (23.2%)	22 (32.8%)	22 (29.7%)		
Metastatic sites					
Brain	33 (25.6%)	14 (20.9%)	11 (14.9%)	χ²=3.22	0.200
Liver	20 (15.5%)	5 (7.5%)	6 (8.1%)	χ²=3.95	0.139
Bone	29 (22.5%)	24 (35.8%)	31 (41.9%)	χ²=9.19	0.010
Immunotherapy regime				χ²=20.60	0.002
Tislelizumab	74 (57.4%)	21 (31.3%)	33 (44.6%)		
Pembrolizumab	25 (19.4%)	9 (13.4%)	12 (16.2%)		
Sintilimab	14 (10.9%)	18 (26.9%)	13 (17.6%)		
Camrelizumab	16 (12.3%)	19 (28.4%)	16 (%)		

χ², Chi-square test; -, Fisher exact; ECOG-PS, Eastern Cooperative Oncology Group performance status; TPS, tumor proportion score.

### 3.2 Efficacy of ICIs plus chemotherapy administered in different sequences

#### 3.2.1 Survival

**Figure 2:**
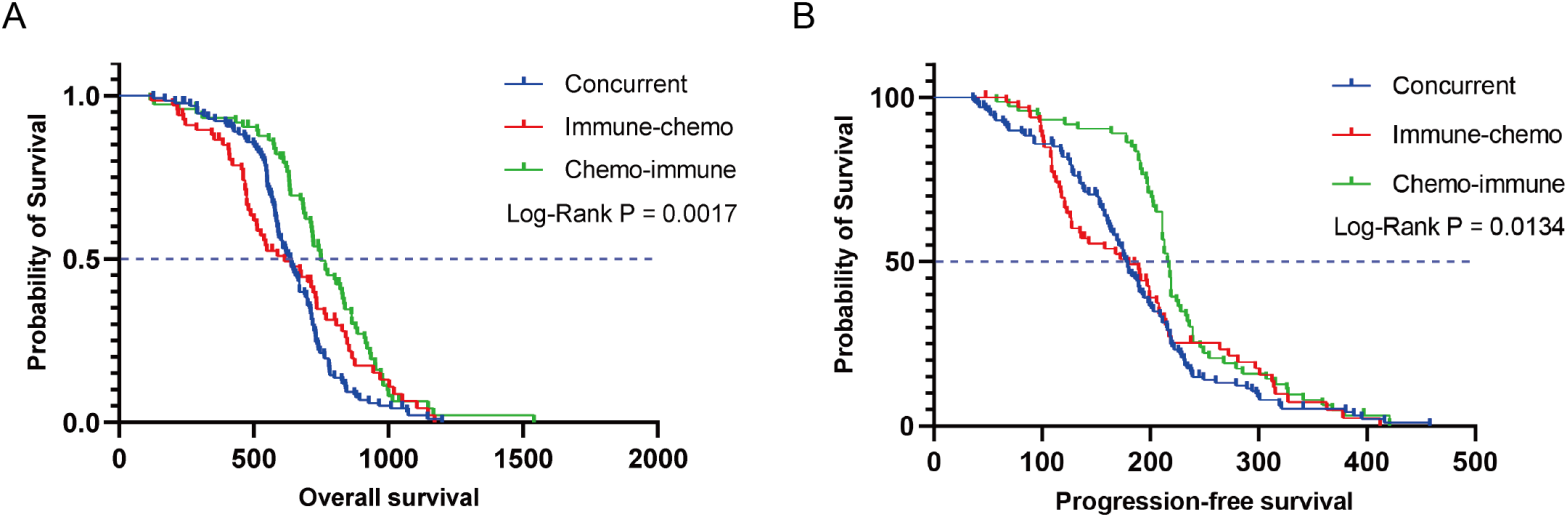
Kaplan-Meier survival curves for OS **(A)** and PFS **(B)** in the concurrent group, immune-chemo group, and chemo-immune group.

In the overall population of NSCLC patients treated with ICIs plus chemotherapy, the median OS (mOS) and median PFS (mPFS) were 672 days (**Supplementary Figure S1A**) and 197 days (**Supplementary Figure S1B**), respectively. Based on the administration sequence of ICIs in combination with chemotherapy, NSCLC patients were stratified into three distinct cohorts. In the concurrent group, immune-chemo group, and chemo-immune group, the mOS was 636, 615, and 749 days, respectively (**Figure 2A**), with a log-rank *P* value of 0.0017. Similarly, mPFS showed distinct patterns at 178 days, 180 days, and 216 days for the respective groups (log-rank *P* = 0.0134) (**Figure 2B**). The chemo-immune group demonstrated a statistically significant improvement in OS compared to concurrent group, with a HR of 0.5650 (95% CI: 0.4241-0.7525; *P* < 0.0001) as illustrated in **Supplementary Figure S1C**. PFS analysis similarly favored the chemo-immunotherapy approach (HR 0.6398, 95% CI: 0.4800-0.8528; *P* = 0.0027; **Supplementary Figure S1D**). In addition, compared with the immune-chemo group, the HRs of OS and PFS in the chemo-immune group were 0.7405 (95% CI: 0.5180 to 1.058, *P* = 0.0069) (**Supplementary Figure S1E**) and 0.6934 (95% CI: 0.4838 to 0.9937, *P* = 0.0006) (**Supplementary Figure S1F**). Collectively, the results indicated that patients in the chemo-immune group have longer OS and PFS compared to the concurrent group and immune-chemo group, with statistically significant differences.

#### 3.2.2 Efficacy

**Figure 3:**
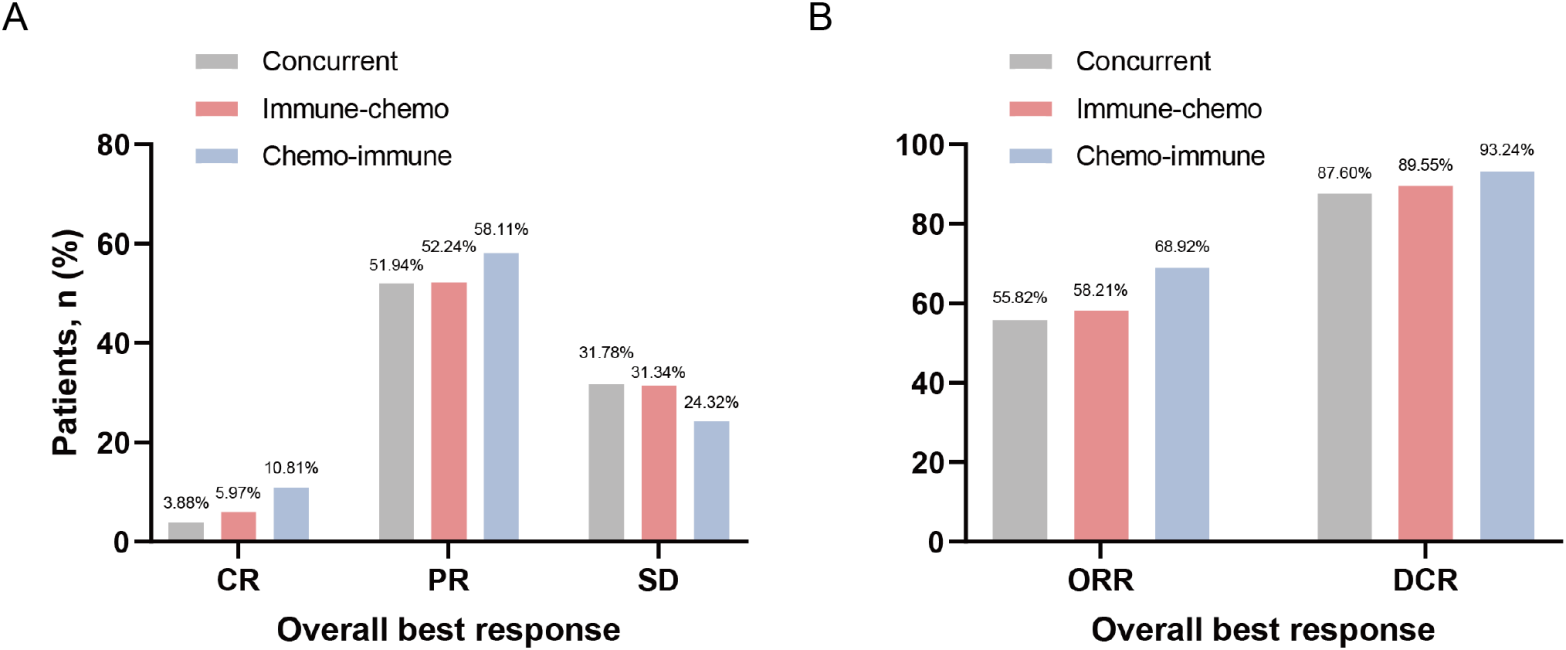
Frequency of the best overall response to ICIs plus chemotherapy regimen. **(A)** Tumor responses in the concurrent group, immune-chemo group, and chemo-immune group. **(B)** ORR and DCR in the concurrent group, immune-chemo group, and chemo-immune group.

In the overall NSCLC population treated with ICIs plus chemotherapy, there were 17 (6.29%), 145 (53.70%), and 80 (29.63%) patients who achieved CR, PR, and SD respectively (**Supplementary Figure S2A**). The ORR and DCR were 60.00% and 89.63%, respectively (**Supplementary Figure S2B**). In the concurrent group, immune-chemo group, and chemo-immune group, the numbers of patients achieving CR were 5 (3.88%), 4 (5.97%), and 8 (10.81%), respectively; PR were 67 (51.94%), 35 (52.24%), and 43 (58.11%), respectively; SD were 41 (31.78%), 21 (31.34%), and 18 (24.32%), respectively (**Figure 3A**). Additionally, the ORR for the concurrent group, immune-chemo group, and chemo-immune group were 72 (55.82%), 39 (58.21%), and 51 (68.92%) patients, respectively, and the DCR were 113 (87.60%), 60 (89.55%), and 69 (93.24%) patients, respectively (**Figure 3B**). There was no statistical difference in efficacy between the three groups.

#### 3.2.3 Subgroup analysis

**Figure 4:**
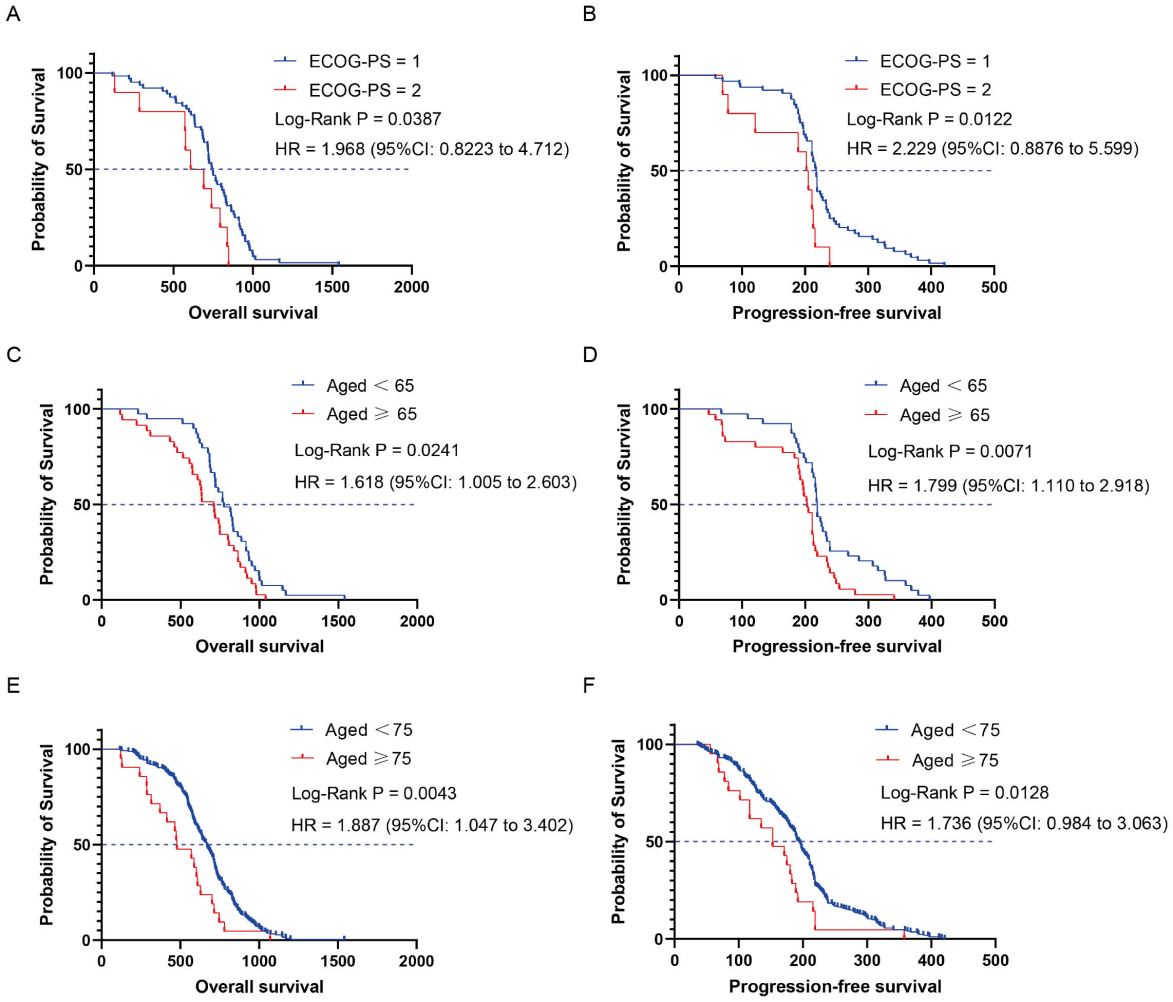
Kaplan-Meier curves depicting survival time endpoints in relation to subgroup analysis of the chemo-immune group. **(A)** OS of ECOG-PS = 1 and ECOG-PS = 2 groups. **(B)** PFS of ECOG-PS = 1 and ECOG-PS = 2 groups. **(C)** OS of aged < 65 and aged ≥ 65 groups. **(D)** PFS of aged < 65 and aged ≥ 65 groups. **(E)** OS of aged < 75 and aged ≥ 75 groups. **(F)** PFS of aged < 75 and aged ≥ 75 groups.

Next, we focused on exploring the population that benefited from the chemo-immune group. Seventy-four advanced NSCLC patients who received chemotherapy followed by ICIs were stratified into different subgroups based on demographics and clinicopathological characteristics (**Supplementary Tables S1**, **S2**). Subgroup analysis revealed that patients with an ECOG-PS score of 1 and 2 had mOS of 745 and 659 days, respectively. Compared with the ECOG-PS = 1 subgroup, the HR for OS in the ECOG-PS = 2 subgroup was 1.968 (95% CI: 0.822 to 4.712, *P* = 0.0387) (**Figure 4A**). The mPFS of patients in the ECOG-PS = 1 and 2 subgroups were 217.5 and 203.5 days, respectively, and the HR for PFS in the ECOG-PS = 2 subgroup compared with the ECOG-PS = 1 subgroup was 2.229 (95% CI: 0.887 to 5.599, *P* = 0.0122) (**Figure 4B**). In addition, age also had a significant impact on the efficacy of ICIs plus chemotherapy regimens. The mOS and mPFS were 774 and 219 days for patients aged < 65 years, while those aged ≥ 65 years were 710 and 203 days, respectively. Compared with the subgroup of patients aged < 65 years, the HR for OS in patients aged ≥ 65 years was 1.618 (95% CI: 1.005 to 2.603, *P* = 0.0241) (**Figure 4C**), and the HR for PFS was 1.799 (95% CI: 1.110 to 2.918, *P* = 0.0071) (**Figure 4D**). Due to the insufficient number of patients over 75 years old in the chemo-immune group, the overall population was used to explore the efficacy of the 75-year-old age subgroup. Compared to the mOS of patients aged < 75 years (669 days), the mOS for patients aged ≥ 75 years was 478 days, with HR of 1.887 (95% CI: 1.047 to 3.402, *P* = 0.0043) (**Figure 4E**). Compared to the mPFS of patients aged < 75 years (194 days), patients aged ≥ 75 years was 153 days, with HR of 1.736 (95% CI: 0.984 to 3.063, *P* = 0.0128) (**Figure 4F**).

### 3.3 Safety of ICIs plus chemotherapy administered in different sequences

**Figure 5:**
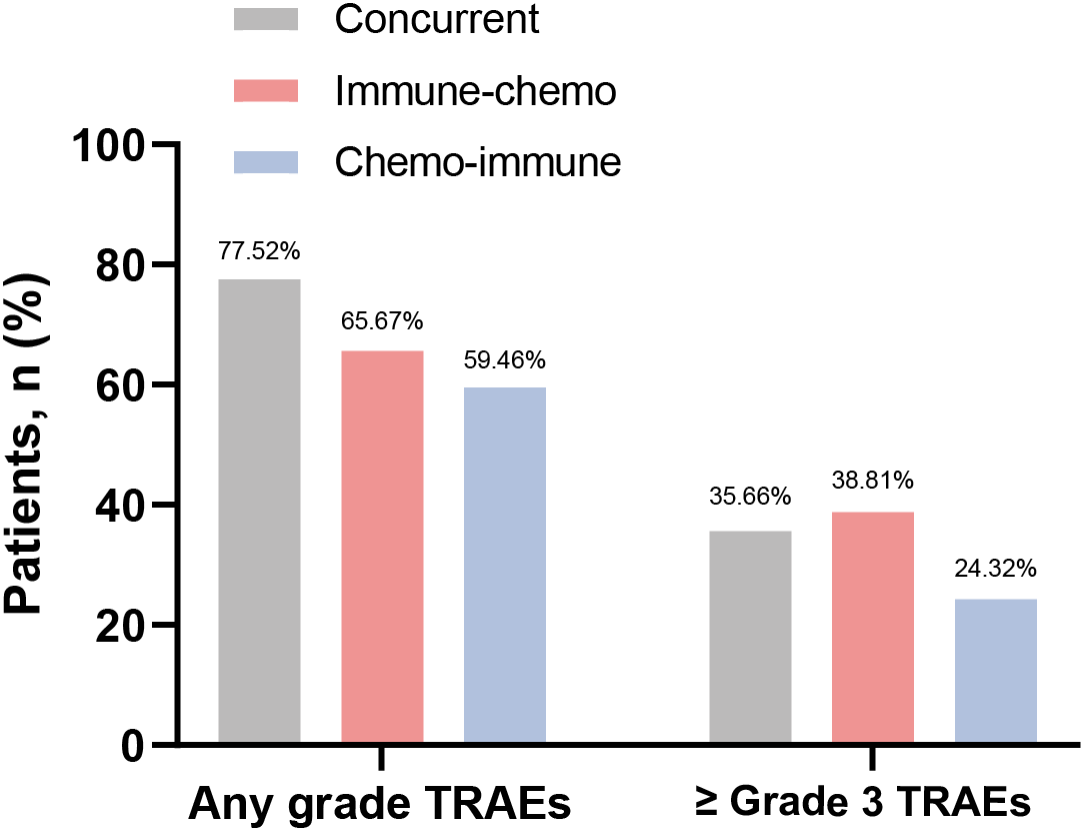
Frequency of any grade TRAEs and Grade 3 or higher TRAEs in the concurrent group, immune-chemo group, and chemo-immune group.

In the total population, the overall incidence of TRAEs of any grade was 69.63% (188/270), with 90 cases (33.33%) experiencing TRAEs of Grade 3 or higher (**Supplementary Figure S2C**). Among the three groups, 77.52% (100/129), 65.67% (44/67), and 59.46% (44/74) TRAEs occurred in the concurrent group, the immune-chemo group, and the chemo-immune group, respectively (**Figure 5**). There was a significant difference in the incidence of TRAE at any grade among the three groups (χ² = 7.91, *P* = 0.019). The number of patients with high-grade (≥ Grade 3) TRAEs in each group was 46 (35.66%), 26 (38.81%), and 18 (24.32%), respectively (**Figure 5**). However, there was no difference in the incidence of high-grade TRAEs between the three groups (χ² = 3.92, *P* = 0.141). In this study, all-grade TRAEs were primarily manifested as treatment-related hematologic toxicity and skin toxicity, while Grade 3 or higher TRAEs mainly involved treatment-related hematologic toxicity and pulmonary toxicity (**Table 3**).

**Table 3:** Treatment-related adverse events of three groups.

	Any grade, n (%)			≥ Grade 3, n (%)		
	Concurrent group	Immune-chemo group	Chemo-immune group	Concurrent group	Immune-chemo group	Chemo-immune group
Treatment-related adverse events	100 (77.52%)	44 (65.67%)	44 (59.46%)	46 (35.66%)	26 (38.81%)	18 (24.32%)
Treatment-related skin toxicity	13 (10.08%)	7 (10.45%)	9 (12.16%)	0	0	0
Treatment-related hematologic toxicity	49 (37.98%)	22 (32.84%)	23 (31.08%)	14 (10.85%)	15 (22.39%)	9 (12.16%)
Treatment-related pulmonary toxicity	9 (6.98%)	2 (2.99%)	2 (2.70%)	11 (8.52%)	6 (8.96%)	5 (6.76%)
Treatment-related gastrointestinal toxicity	12 (9.30%)	3 (4.48%)	5 (6.76%)	4 (3.10%)	1 (1.49%)	1 (1.35%)
Treatment-related neurotoxicity	4 (3.10%)	1 (1.49%)	0	6 (4.65%)	2 (2.98%)	1 (1.35%)
Treatment-related endocrine toxicity	10 (7.75%)	6 (8.95%)	5 (6.76%)	0	0	0
Treatment-related myocardial toxicity	3 (2.33%)	3 (4.48%)	0	11 (8.53%)	2 (2.98%)	2 (2.70%)

Grade according to the Common Terminology Criteria for Adverse Events version 4.0.

### 3.4 TRAEs were associated with a favorable survival prognosis

**Figure 6:**
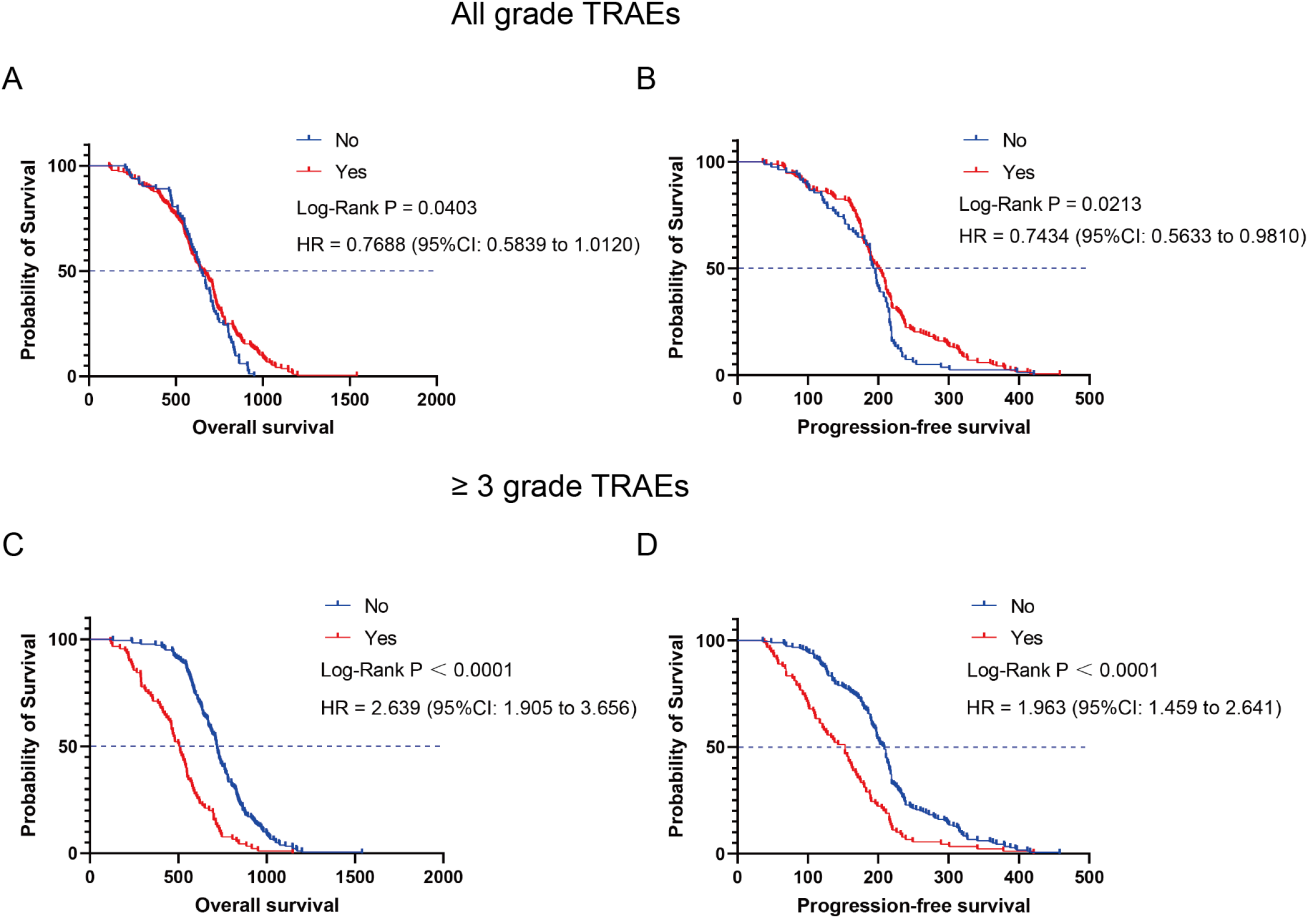
Kaplan-Meier curves depicting survival time in relation to the presence or absence of TRAEs. **(A)** OS of TRAEs group and non-TRAEs group. **(B)** PFS of TRAEs group and non-TRAEs group. **(C)** OS of high-level TRAEs (≥ Grade 3) group and non-high-level TRAEs group. **(D)** PFS of high-level TRAEs (≥ Grade 3) group and non-high-level TRAEs group.

**Figure 7:**
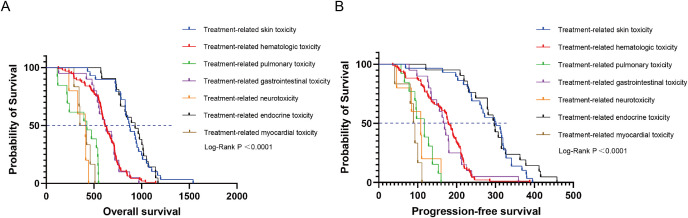
Kaplan-Meier curves depicting survival time endpoints in relation to subgroup analysis of TRAEs group. **(A)** OS in patients with TRAEs stratified by different sites of occurrence. **(B)** PFS in patients with TRAEs stratified by different sites of occurrence.

All patients were categorized into the TRAEs group (n = 188) and the non-TRAEs group (n = 82) based on the occurrence of TRAEs. Compared with the non-TRAEs group, the TRAEs group had longer mOS (644 days vs. 654.5 days, *P* = 0.0403, HR = 0.7688 [95% CI: 0.5839 to 1.0120]) (**Figure 6A**) and mPFS (194.5 days vs. 200 days, *P* = 0.0213, HR = 0.7434 [95% CI: 0.5633 to 0.9810]) (**Figure 6B**). The results of this study suggested that the occurrence of TRAEs in NSCLC patients may herald an improved survival prognosis. However, patients who developed Grade 3 and higher TRAEs had an extremely poor prognosis. We categorized patients into high-level TRAEs group (n = 90) and non-high-level TRAEs group (n = 180) according to the occurrence of high-level (≥ Grade 3) TRAEs. The mOS was 507 and 718 days, and the mPFS was 153 and 208.5 days for the high-level TRAEs group and the non-high-level TRAEs group, respectively. Compared with the non-high-level TRAEs group, the HR for OS in the high-level TRAEs group was 2.639 (95% CI: 1.905 to 3.656, *P* < 0.0001) (**Figure 6C**), and the HR for PFS was 1.963 (95% CI: 1.459 to 2.641, *P* < 0.0001) (**Figure 6D**). Depending on the site of TRAEs occurrence, we conducted subgroup analyses of NSCLC patients with TRAEs. The results showed a significant increase in mOS (874 or 932 days) and mPFS (301 or 297 days) when patients experienced treatment-related skin toxicity or treatment-related endocrine toxicity (**Figure 7**). Additionally, patients with high-grade treatment-related hematologic toxicity had better survival compared to those with other high-grade TRAEs (**Supplementary Figure S3**).

## 4 Discussion

Although the high anti-tumor efficacy of ICIs plus chemotherapy has been widely demonstrated in clinical practice, the administration sequence of the two treatments remains a mystery. Currently, the sequence of ICIs plus chemotherapy in phase III RCTs is administered on the same day (13). However, this may not be the optimal sequence. Our study based on real world statistics indicated that the current sequence of ICIs plus chemotherapy could be further optimized. Administering chemotherapy before ICIs, rather than concurrent use, may have better improved OS, PFS, and ORR in advanced NSCLC patients (**Supplementary Figure S4**).

Chemotherapy administered before ICIs yielding superior outcomes can be explained by following reasons. Firstly, chemotherapy can induce immunogenic cell death, thereby activating the adaptive immune response and upregulating antigen presentation mechanisms, which enhances the effectiveness of subsequent ICIs treatment (19–21). Secondly, chemotherapy can improve T-cells initiation by reducing tumor burden (22). Lastly, T-cells toxicity can be avoided by administering chemotherapy before ICIs treatment (23, 24). Therefore, it is recommended to use chemotherapy preemptively and to administer ICIs after the peak blood concentration of chemotherapy drugs has passed to minimize the cytotoxic impact on T cells and to maximize anti-tumor efficacy.

Indeed, several clinical studies support the notion that administering chemotherapy before ICIs results in better therapeutic outcomes. In a phase II RCT involving 30 patients with locally advanced esophageal cancer, the sequence of chemotherapy and anti-PD-1 antibody treatment was explored to assess its impact on efficacy (25). The study found that there was a higher rate of pathological complete response (pCR) when the interval between ICIs and chemotherapy was more than 48 hours. And administering anti-PD-1 antibody after chemotherapy may be more conducive to synergistic effects. Furthermore, a real-world retrospective study demonstrated that the optimal timing for administering anti-PD-1 antibody after chemotherapy was within 3 to 5 days (26).

In addition, subgroup analysis revealed that chemo-immunotherapy was less effective in patients with high ECOG-PS scores (≥ 2) and in elderly patients (≥ 65 years), particularly those aged 75 and above. This is consistent with the conclusions of our group’s previous meta-analysis studies, which included data from public databases and RCTs (27). Higher ECOG-PS scores indicate poorer general condition in tumor patients, and such individuals often experience inferior survival benefits when receiving anti-tumor therapy. Due to immunosenescence, elderly tumor patients develop an immunosuppressive microenvironment, which impairs the normal functioning of immune cells and affects the efficacy of ICIs.

In this retrospective study, we found that the development of TRAEs of any grade was significantly associated with better survival outcomes in patients treated with ICIs plus chemotherapy. Patients who experienced TRAEs had significantly longer OS and PFS compared with the group without TRAEs. However, the occurrence of Grade 3 or higher TRAEs was associated with a markedly poor prognosis. Multiple studies are in agreement with our conclusions. A multicenter cohort study suggested that the occurrence of clinically meaningful TRAEs was associated with longer OS in locally advanced or metastatic NSCLC (28). Furthermore, a multicenter retrospective study concluded that the earlier occurrence of low-grade TRAEs (< Grade 3) was associated with a better prognosis, while the earlier occurrence of high-grade TRAEs (≥ Grade 3) was associated with a poorer prognosis (29). During the anti-tumor process of ICIs, activated T cells not only target tumor cells but also damage normal tissue cells, leading to the occurrence of TRAEs. The occurrence of TRAEs during anti-tumor therapy typically indicates high T-cell activation, thereby enabling more effective anti-tumor responses.

Based on the varying sites of TRAEs, the subgroup analysis indicated that patients who developed skin toxicity or endocrine toxicity had significantly longer survival outcomes. We considered that skin or endocrine toxicities typically occur early in treatment and rarely result in Grade 3 or higher TRAEs, and patients generally have a favorable survival prognosis. Several studies support this observation. A real-world observational study revealed that pan-cancer patients with skin toxicities or blood toxicities had achieved a longer PFS, than those without corresponding toxicities, respectively (30). For NSCLC, a retrospective study found that the occurrence of skin toxicity or endocrine toxicity was associated with longer PFS and OS (31). Multiple systematic reviews and meta-analyses have found that NSCLC patients treated with ICIs who had endocrine or skin TRAEs tend to predict better prognosis (32–34). It’s unfortunate that currently, there is only clinical evidence available, with no basic research exploring the specific mechanisms involved. We hope that answers will emerge in the future.

Although the results are promising, the limitations of this study need to be acknowledged. There is no clear conclusion on the optimal interval duration for the chemo-immune regimen, which restricts the clinical application of this protocol. To further validate these results and optimize the clinical application of ICIs plus chemotherapy in NSCLC patients, prospective studies with larger sample sizes and more standardized protocols are necessary in the future.

## 5 Conclusion

In summary, our study demonstrated that pre-treatment with chemotherapy before ICIs was associated with improved OS, PFS, and ORR in patients with advanced NSCLC. Additionally, patients who developed any grade of TRAEs during the treatment had better survival outcomes, particularly those with skin or endocrine toxicity.

## Data Availability

The raw data supporting the conclusions of this article will be made available by the authors, without undue reservation.
