# Supplementary material for: The administration sequences of immune checkpoint inhibitors and chemotherapy cause discrete efficacy when treating non-small cell lung cancer: a retrospective study
Source: Front Immunol. 2025 Apr 28;16:1579420. doi: 10.3389/fimmu.2025.1579420 (PMC12066507; doi:10.3389/fimmu.2025.1579420)
Supplement: Supplementary file 1 [file Table1.docx]

*Supplementary Materials*

**Supplementary Table 1.** Survival analysis of OS in different subgroups of patients in the chemo-immune group

| Characteristics | mOS (days) | HR (95% CI) | *P* value |
| --- | --- | --- | --- |
| Male | 728.5 | 1.181 (0.728-1.915) | 0.5093 |
| Female | 770 |  |  |
| Age < 65 | 774 | 1.618 (1.005-2.603) | 0.0241 |
| Age ≥ 65 | 710 |  |  |
| Age < 75 | 669 | 1.887 (1.047-3.402) | 0.0043 |
| Age ≥ 75 | 478 |  |  |
| ECOG-PS = 1 | 745 | 1.968 (0.822-4.712) | 0.0387 |
| ECOG-PS = 2 | 659 |  |  |
| Adenocarcinoma | 766 | 0.942 (0.579-1.532) | 0.7987 |
| Squamous carcinoma | 710 |  |  |
| Never-smokers | 766 | 1.437 (0.856-2.412) | 0.1300 |
| Former or current smokers | 716 |  |  |
| Brain metastasis | 684 | 1.006 (0.529-1.911) | 0.9857 |
| Non-brain metastasis | 742 |  |  |
| Liver metastasis | 873 | 0.873 (0.397-1.923) | 0.7470 |
| Non-liver metastasis | 730.5 |  |  |
| Bone metastasis | 684 | 1.596 (0.976-2.611) | 0.0394 |
| Non-bone metastasis | 774 |  |  |

mOS: median overall survival; HR: hazard ratio; CI: confidence interval; ECOG-PS: Eastern Cooperative Oncology Group performance status;

**Supplementary Table 2.** Survival analysis of PFS in different subgroups of patients in the chemo-immune group

| Characteristics | mPFS (days) | HR (95% CI) | *P* value |
| --- | --- | --- | --- |
| Male | 211 | 1.381 (0.861-2.214) | 0.1890 |
| Female | 221.5 |  |  |
| Age < 65 | 219 | 1.799 (1.110-2.918) | 0.0071 |
| Age ≥ 65 | 203 |  |  |
| Age < 75 | 194 | 1.736 (0.984-3.063) | 0.0128 |
| Age ≥ 75 | 153 |  |  |
| ECOG-PS = 1 | 217.5 | 2.229 (0.887-5.599) | 0.0122 |
| ECOG-PS = 2 | 203.5 |  |  |
| Adenocarcinoma | 213 | 1.094 (0.680-1.760) | 0.7064 |
| Squamous carcinoma | 216 |  |  |
| Never-smokers | 218 | 1.119 (0.685-1.829) | 0.6335 |
| Former or current smokers | 211 |  |  |
| Brain metastasis | 203 | 0.963 (0.512-1.813) | 0.9028 |
| Non-brain metastasis | 216 |  |  |
| Liver metastasis | 226.5 | 0.737 (0.352-1.540) | 0.4599 |
| Non-liver metastasis | 213 |  |  |
| Bone metastasis | 213 | 1.075 (0.676-1.712) | 0.7506 |
| Non-bone metastasis | 217 |  |  |

mPFS: median progression-free survival; HR: hazard ratio; CI: confidence interval; ECOG-PS: Eastern Cooperative Oncology Group performance status;

**Supplementary Figure 1**

**
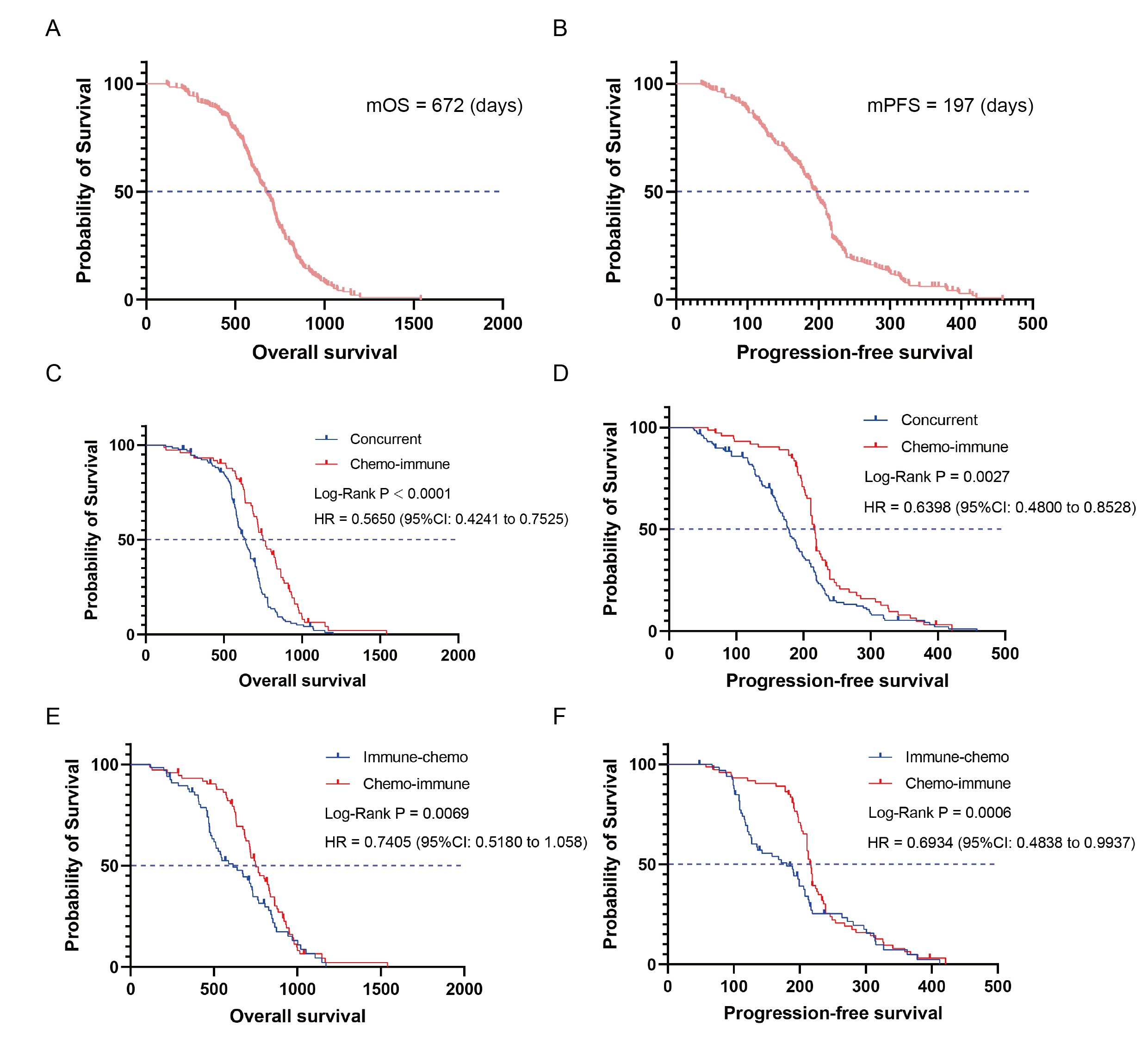
**

**Supplementary Figure 1.** Kaplan-Meier curves depicting survival time endpoints in patients treated with ICIs plus chemotherapy treatment. **(A)** OS in overall NSCLC patients. **(B)** PFS in overall NSCLC patients. **(C)** OS of the concurrent group and chemo-immune group. **(D)** PFS of the concurrent group and chemo-immune group. **(E)** OS of immune-chemo group and chemo-immune group. **(F)** PFS of immune-chemo group and chemo-immune group.

**Supplementary Figure 2**

**
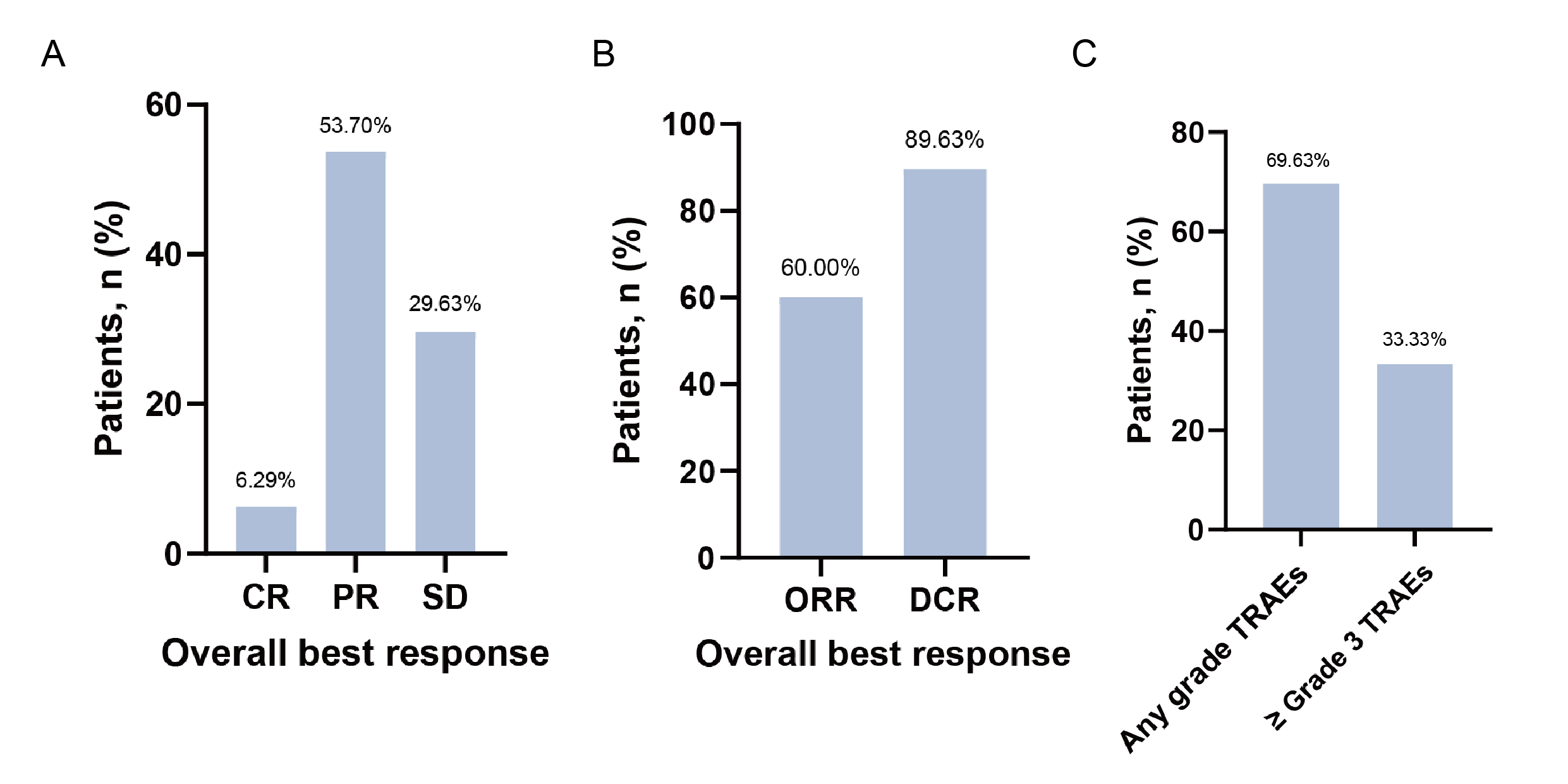
**

**Supplementary Figure 2.** Frequency of the best overall response and the incidence of TRAEs in overall NSCLC patients. **(A)** Tumor responses in overall NSCLC patients. **(B)** ORR and DCR in overall NSCLC patients. **(C)** Any grade and Grade 3 or higher TRAEs in overall NSCLC patients.

**Supplementary Figure 3**

**
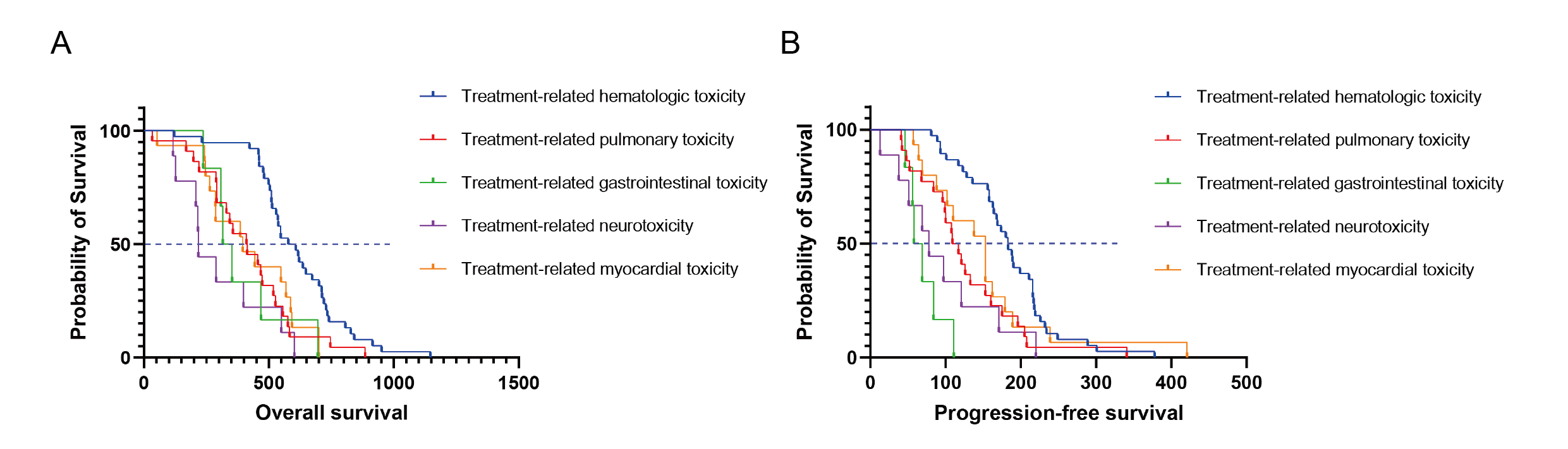
**

**Supplementary Figure 3.** Kaplan-Meier curves depicting survival time endpoints in relation to subgroup analysis of high-level TRAEs (≥ Grade 3) group. **(A)** OS in patients with high-level TRAEs stratified by different sites of occurrence. **(B)** PFS in patients with high-level TRAEs stratified by different sites of occurrence.

**Supplementary Figure 4**


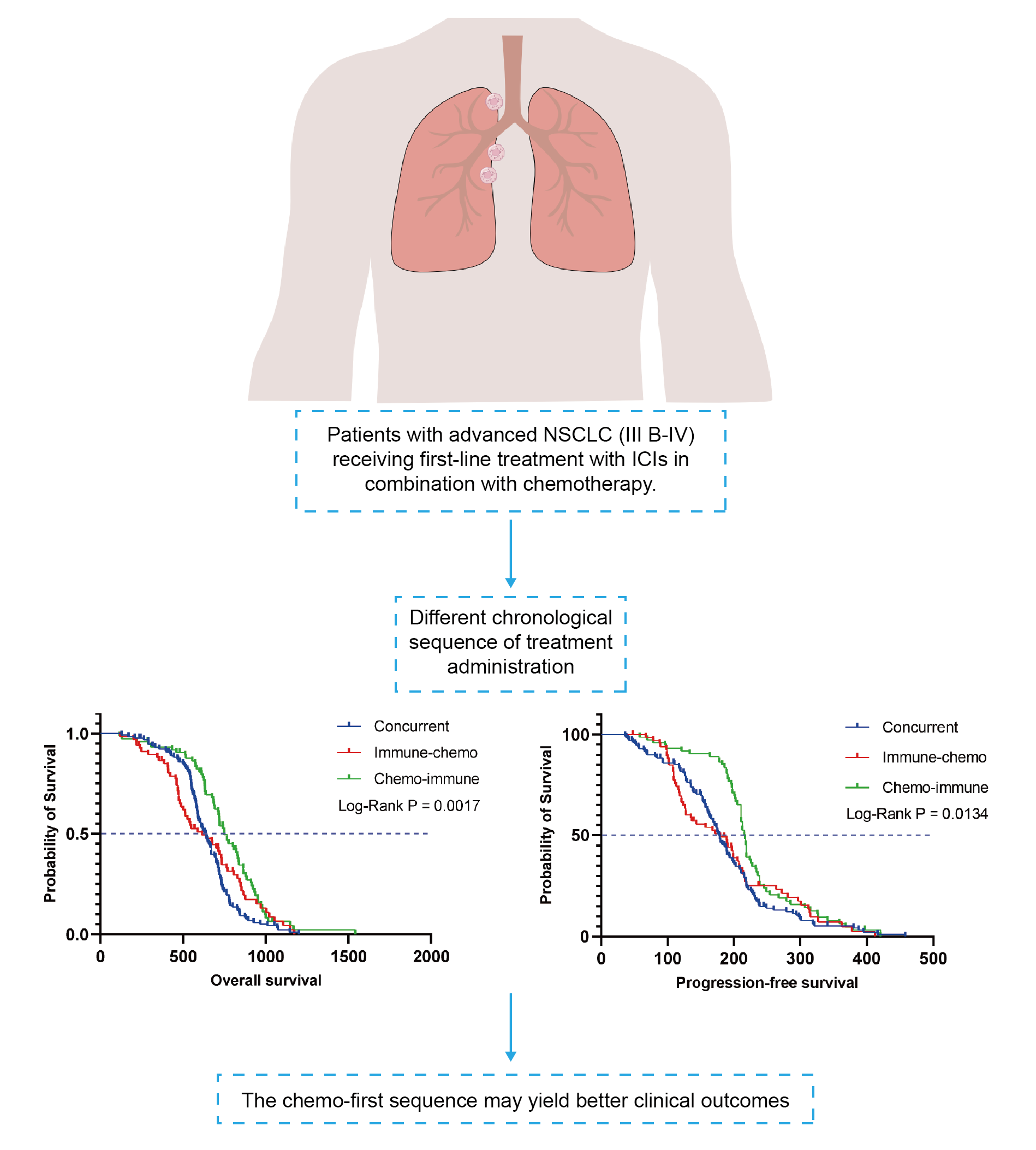


**Supplementary Figure 4.** Graphical abstract of this study.
